# Preparation of Microporous Polypropylene/Titanium Dioxide Composite Membranes with Enhanced Electrolyte Uptake Capability via Melt Extruding and Stretching

**DOI:** 10.3390/polym9030110

**Published:** 2017-03-17

**Authors:** Shan Wang, Abdellah Ajji, Shaoyun Guo, Chuanxi Xiong

**Affiliations:** 1 State Key Laboratory of Advanced Technology for Materials Synthesis and Processing, School of Materials Science and Engineering, Wuhan University of Technology, Wuhan 430070, China; shanwang@whut.edu.cn; 2 CREPEC, Chemical Engineering Department, Ecole Polytechnique, Montreal, QC H3C 3A7, Canada; abdellah.ajji@polymtl.ca; 3 The State Key Laboratory of Polymer Materials Engineering, Polymer Research Institute of Sichuan University, Chengdu 610065, China; sguo@scu.edu.cn

**Keywords:** polypropylene, microporous membrane, titanium dioxide, lamellar orientation, stretching, electrolyte uptake

## Abstract

In this work, a blending strategy based on compounding the hydrophilic titanium dioxide (TiO_2_) particles with the host polypropylene (PP) pellets, followed by the common membrane manufacture process of melt extruding/annealing/stretching, was used to improve the polarity and thus electrolyte uptake capability of the PP-based microporous membranes. The influence of the TiO_2_ particles on the crystallinity and crystalline orientation of the PP matrix was studied using differential scanning calorimetry (DSC), X-ray diffraction (XRD), and infrared dichroic methods. The results showed that the TiO_2_ incorporation has little influence on the oriented lamellar structure of the PP-based composite films. Investigations of the deformation behavior indicated that both the lamellar separation and interfacial debonding occurred when the PP/TiO_2_ composite films were subjected to uniaxial tensile stress. The scanning electron microscopy (SEM) observations verified that two forms of micropores were generated in the stretched PP/TiO_2_ composite membranes. Compared to the virgin PP membrane, the PP/TiO_2_ composite membranes especially at high TiO_2_ loadings showed significant improvements in terms of water vapor permeability, polarity, and electrolyte uptake capability. The electrolyte uptake of the PP/TiO_2_ composite membrane with 40 wt % TiO_2_ was 104%, which had almost doubled compared with that of the virgin PP membrane.

## 1. Introduction

In recent years, microporous polypropylene (PP) membranes have been widely used as separators for fabricating lithium batteries [1,2,3,4,5]. These separators can guarantee the rapid transport of the ionic charge carriers and keep the negative and positive electrodes apart to avoid an internal short [2]. The nonpolar properties of PP can offer excellent chemical stability, mechanical strength, and other advantages; however, they also lead to extremely low wettability with liquid electrolytes such as ethylene carbonate (EC) and propylene carbonate (PC), which consequently adversely affects battery performance [3,4]. Therefore, it is of much significance to develop novel PP membranes with higher polarity and thus an enhanced ability to uptake and retain the polar electrolyte.

To achieve this goal, different strategies have been carried out to improve the membrane surface, i.e., increasing the amount of surface polar groups and maximizing hydration. These methods include plasma treatment, surface coating with hydrophilic materials, radical grafting hydrophilic functional groups, surface polymerization of hydrophilic monomers, and so on [6,7,8,9,10,11,12,13,14,15,16]. However, some inherent drawbacks of these methods have been recognized. For example, temporary hydrophilicity is an unavoidable problem in the cases of plasma treatment and surface coating; radical grafting and/or polymerization methods are often complicated and require additional steps in the membrane preparation, thus increasing the cost [17,18]. Accordingly, the above techniques are greatly limited in applications on an industrial scale.

Very recently, a blending strategy, i.e., physically mixing the host polymer with a hydrophilic modifier, subsequently followed by the conventional membrane fabrication process, is proposed and considered as a potentially promising method for manufacturing the polyolefin membranes with enhanced polarity, due to its feasible, large-scale, and cost-effective characteristics [17,18,19]. Commonly, the fabrication of PP microporous membranes involves the melt extrusion of precursor films followed successively by the high-temperature annealing and cold and hot stretching procedures [20,21,22,23,24,25,26]. It has been well established that the generation of a row nucleated lamellar structure in precursor films plays a key role in determining the porous structure and thus the permeability performance of PP membranes [20,21,22]. Thereby, the key point of the blending strategy is to reduce the influence of the modifier on the crystalline morphology of the PP precursor film as much as possible. Saffar et al. have developed the hydrophilic PP-based membranes from the blends of PP and PP-grafted maleic anhydride (PP-*g*-MAH) or acrylic acid (PP-*g*-AA) using a commercial drying process [18]. The results showed that a 2 wt % modifier was optimal for membranes to realize good hydrophilicity while causing minimal change to the crystalline structure compared to the neat PP film.

In comparison with their counterparts of hydrophilic polymers or oligomers, modifiers of hydrophilic inorganic fillers possess some extra advantages in terms of thermal, mechanical, and electrical properties [27,28]. However, to the best of our knowledge, little work has been carried out to investigate the influence of inorganic fillers on the structure and properties of PP membranes. In this work, hydrophilic titanium dioxide (TiO_2_) particles of submicron size were chosen and compounded with host PP pellets at different ratios to improve the wettability and retention of the liquid electrolytes. The influence of the TiO_2_ particles on the crystalline structure and deformation behavior of PP-based precursor films obtained by melt extrusion was systematically investigated. Then, the PP/TiO_2_ composite films were annealed and cold- and hot-stretched into microporous membranes. The porous structure, water vapor permeability, water contact angle, electrolyte uptake, and mechanical properties of the PP/TiO_2_ composite membranes were thus comprehensively studied.

## 2. Materials and Methods

### 2.1. Materials

A commercial polypropylene homopolymer (Pro-fax 6523) purchased from LyondellBasell Industries (Rotterdam, The Netherlands) was chosen as the polymer matrix. The resin has a density of 0.9 g/cm^3^ and a melt flow rate (MFR) value of 4.0 g/10 min (230 °C, 2.16 kg). Titanium dioxide (TiO_2_, Ti-Pure R-960) from DuPont Company (Wilmington, DE, USA) were used as the filler. According to the manufacture, the particles have an average size of 0.50 μm.

### 2.2. Film and Membrane Preparation

PP/TiO_2_ composites with different TiO_2_ loadings were prepared with an intermeshing co-rotating twin screws extruder (Leistritz Corporation, Allendale, MI, USA, ZSE 18 HPe). A temperature profile ranging from 160 to 200 °C was set along the extruder, and the screw speed was 60 rpm. The extruded strands were quenched by cold water and subsequently pelletized and dried for the following film extrusion.

To prepare the precursor films, cast extrusions of the pre-dried PP/TiO_2_ composites were performed using a 45 mm single extruder (Killion Corp., Indianapolis, IN, USA) equipped with a slit die (a width of 25 cm and a thickness of 0.8 mm) and a set of cooling rolls. The screw speed was 12 rpm, and the die and roller temperatures were 220 °C and 50 °C, respectively. A draw ratio of 30 (ratio of the roll speed to the die exit velocity) was used to produce films with a thickness of around 27 μm. An air knife was applied right at the die exit to supply air to the film surface for cooling. The virgin PP film was also made as a control using the same processing condition.

The as-prepared precursor films were cut into a 70 mm × 80 mm rectangular shape along the extrusion direction, and were annealed at 145 °C for 10 min in a hot oven. Then, the annealed samples were cold- and hot-stretched into microporous membranes using an Instron machine (ElectroPlus E3000, Instron, Canton, MA, USA) equipped with an environmental chamber. The cold stretching was performed at 25 °C with an extension ratio of 35% at a speed of 100 mm/min, while the hot stretching was performed at 130 °C with an extension ratio of 60% at a speed of 50 mm/min. The as-obtained stretched membranes were subsequently annealed at 135 °C for 90 s to fix the porous structure.

### 2.3. Characterization

Crystallinity (*X*_c_) of the precursor films were analyzed using a TA Instruments Q1000 differential scanning calorimeter (DSC, TA Instruments, New Castle, DE, USA). Samples were heated from 40 to 200 °C at a heating rate of 10 °C/min. *X*_c_ was calculated using a fusion heat of 209 J/g for fully crystalline PP [29].

X-ray diffraction (XRD) patterns of the films were recorded with a Philips X’Pert-MRD diffractometer (PANalytical, Almelo, The Netherlands) using Ni-filtered Cu Kα radiation (λ = 0.154 nm). The spectra were collected in the diffraction angle (2θ) range of 5°–32°.

Infrared dichroic properties of the films were measured using a Spectrum 65 Fourier transform infrared spectrometer (PerkinElmer Corp., Waltham, MA, USA) equipped with a wire grid polarizer. For each specimen, two polarized spectra, namely parallel and perpendicular to the extrusion direction, were collected with 64 scans at a resolution of 4 cm^−1^ in the wavenumber range of 4000–600 cm^−1^. The orientation factor (*f*) is defined as follows:

*f* = (*D* − 1)/(*D* + 2) (1)

*D* = *A*_//_/*A*_⏊_ (2)

where *A*_//_ and *A*_⏊_ are the absorbance parallel and perpendicular to the extrusion direction, respectively. For PP, absorbance at the wavenumber of 998 cm^−1^ was chosen to calculate the orientation of the crystalline phase [21].

The tensile tests were carried out using an Instron 3365 machine (Instron, Canton, MA, USA) at room temperature. The initial grip distance was set to 50 mm. Films with a 25 mm width were stretched along the flow direction at a speed of 100 mm/min.

The surface and cross-section morphologies of the precursor films and membranes were examined by a field emission scanning electron microscope (FESEM-Hitachi S4700, Tokyo, Japan) at an accelerating voltage of 2 kV. To prepare the cross section, the specimen was immersed into the epoxy followed by an ultrathin sectioning in liquid nitrogen. All the samples were gold-coated for 15 s before observations.

The water vapor transmission rates (WVTR) of the membranes were measured using a MOCON PERMATERAN-W Model 101 K at 37.8 °C. Before measurement, calibration of the cell was performed with the relative humidity kept around 60% in the lower chamber.

The water contact angle (WCA) of the microporous membrane was determined by the sessile drop method using a VCA Optima machine (AST Products, Inc., Billerica, MA, USA) with a precision camera and advanced PC technology. During testing, a 1 μL droplet was lowered onto the membrane surface with a microsyringe and equilibrated for 10 s before WCA observation. Each reported WCA was an average of at least five measurements.

To measure the electrolyte uptake, the stretched microporous membrane was soaked in the liquid electrolyte for 12 h, and the ratio of the weight gain compared to the dry membrane was calculated according to expression Uptake (%) = (*W* − *W*_0_)/*W*_0_ × 100%, where *W*_0_ and *W* are the weights of the membrane before and after absorbing the liquid electrolyte, respectively.

## 3. Results and Discussion

### 3.1. Structures of PP/TiO_2_ Composite Films

**Figure 1:**
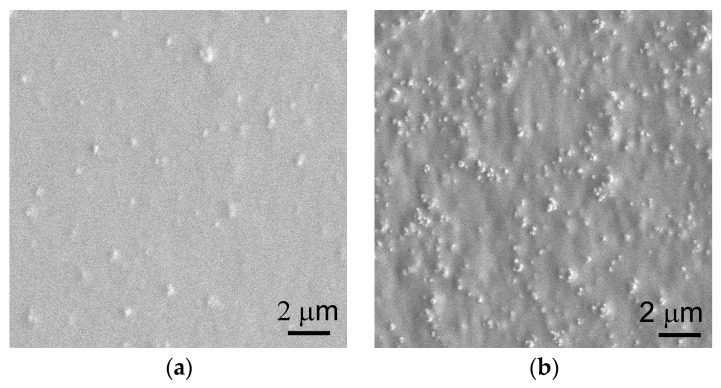
Surface morphologies of PP/TiO_2_ composite films with the TiO_2_ content of (**a**) 10 wt % and (**b**) 40 wt %.

PP/TiO_2_ composite films were melt extruded using a draw ratio of 30, the same as that for the virgin PP film. Unfortunately, continuously cast extrusion and acquisition of flat and uniform PP/TiO_2_ composite films failed when the TiO_2_ content reached 50 wt % (i.e., 13.3 vol %). This can be well understood since the drawability of the composite melts decreases sharply when filler loading approaches a threshold value, which is typically about 16 vol % for the spherical particles [30]. In this regard, the TiO_2_ content is controlled within 40 wt % in this study. Figure 1a,b present the SEM surface morphologies of the PP/TiO_2_ composite films with 10 and 40 wt % TiO_2_, respectively. It can be observed that the TiO_2_ particles are homogeneously dispersed in the PP matrix, even at a high loading of 40 wt %. The amount of the particles located on the surface increases and their distance decreases with increased TiO_2_ content. It is speculated that, in addition to the selection of appropriate submicron-sized TiO_2_ particles that possess a relatively low surface energy, the elongation flow during the cast extrusion process also helps to reduce the possibility of particle agglomeration. Considering that PP/TiO_2_ microporous membranes are fabricated by uniaxial stretching their precursors, a good dispersion of TiO_2_ particles will benefit an evenly distributed stress in the stretched films and thus a uniform pore distribution in the obtained membranes.

It has been known that PP film having a large quantity of oriented lamellae greatly favors the formation of voids when subjected to uniaxial stretching [20,21]. With this regard, crystallinity and crystalline orientation are considered as two key parameters for evaluating the pore formation capabilities of the PP-based precursor films. Table 1 lists the melting temperature (*T*_m_) and crystallinity (*X*_c_) of the PP and PP/TiO_2_ composite films analyzed by DSC. For the virgin PP film, the *T*_m_ and *X*_c_ are 164.4 °C and 41.8%, respectively. It is found that both the *T*_m_ and *X*_c_ for the PP/TiO_2_ composite films with different TiO_2_ loadings do not show significant variations compared to those of the virgin PP film. This may suggest that TiO_2_ particles have little influence on the crystallization of the PP matrix during the extrusion process wherein the elongational flow is applied.

**Figure 2:**
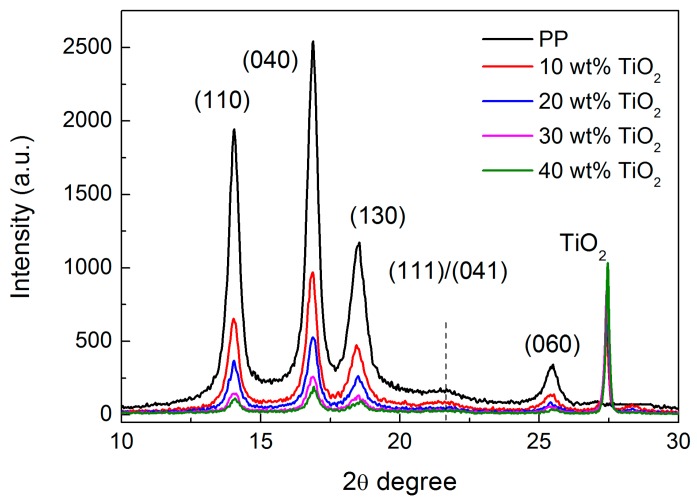
X-ray diffraction patterns for the PP and PP/TiO_2_ composite films with different TiO_2_ concentrations.

Figure 2 shows the XRD results for the PP and PP/TiO_2_ composite films. Distinct peaks centered at the 2θ angles of 14.0°, 16.9°, 18.5°, and 25.4° can be observed for the virgin PP film, corresponding to the α-form crystallographic planes (110), (040), (130), and (060), respectively; however, diffraction peaks of the (111/041) planes (2θ = 21.3°, 21.8°), which are also the characteristics of the α-form PP, are very weak [31]. This behavior has been previously reported in the case of an oriented PP sample, suggesting that the specimen possesses a highly oriented lamellar structure in terms of its crystalline morphology [32]. Compared to the virgin PP film, no distinctive difference in the diffraction angles is found for the PP/TiO_2_ composite films, except for an additional peak appearing at around 27°, which is a characteristic reflection of the TiO_2_ particles [33], indicating that the incorporation of the TiO_2_ particles does not change the crystal form of the PP matrix. Moreover, the diffraction peak of (111/041) planes is still relatively weak compared to other diffraction peaks for these composite films. Consequently, it is speculated that the addition of TiO_2_ particles has little effect on the crystal structure of the PP matrix, and the PP/TiO_2_ composite films may still possess an orientated lamellar structure.

**Figure 3:**
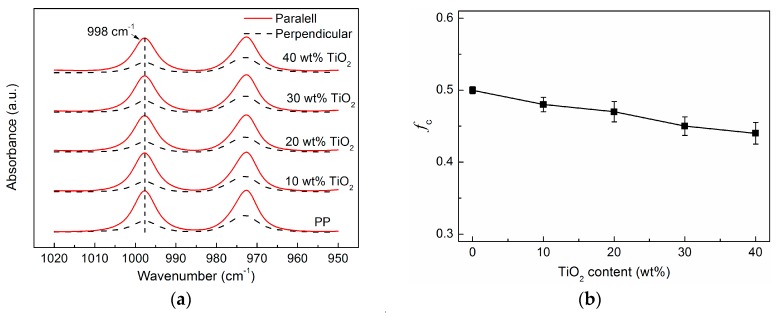
(**a**) Polarized IR spectra for the PP and PP/TiO_2_ composite films in the range of 1020 to 950 cm^−^^1^. (**b**) Crystalline orientation *(f*_c_) of PP as function of TiO_2_ content.

To quantitatively analyze the influence of TiO_2_ particles on the PP crystalline orientation, infrared dichroic properties of the PP/TiO_2_ composite films were studied. As clearly shown in Figure 3a, the absorbance at band 998 cm^−1^ ascribed to the crystalline vibration in the parallel direction is much stronger than that in the perpendicular direction for all the films, suggesting the presence of PP crystalline orientation (*f*_c_) along the extrusion direction. Based on the variations of the band strength at 998 cm^−1^ in the two directions, *f*_c_ was calculated as shown in Figure 3b. It is seen that *f*_c_ decreases slightly from 0.50 to 0.44 as the TiO_2_ loading increases from 0 to 40 wt %. This is contrary to the common results for the particulate-filled polymer composites, for which the oriented crystallization would be seriously disturbed, even when adding a small amount of spherical particles [34,35]. It is thus suggested that the heterogeneous nucleation effect of TiO_2_ particles on PP is negligible compared to the effect of elongation-induced crystallization during the cast extrusion process. Previous studies have demonstrated that row-nucleated lamellar structure is characteristic of the crystalline morphology for PP cast films when crystalline orientation is more than 0.3 [20]. Therefore, it is reasonably inferred that all PP/TiO_2_ composite films in this study dominantly possess a row-nucleated lamellar structure similar to that in the virgin PP film.

### 3.2. Deformation Behavior of PP/TiO_2_ Composite Films

**Figure 4:**
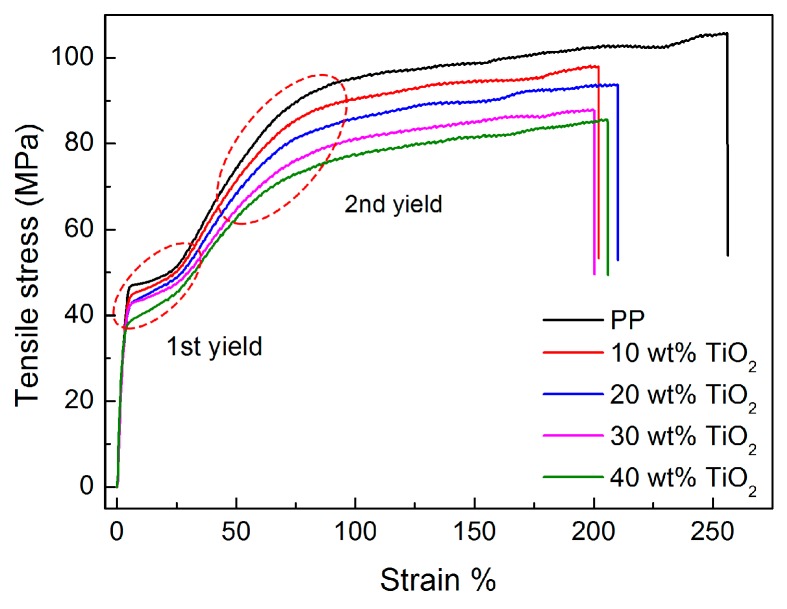
Stress–strain curves of the annealed PP and PP/TiO_2_ composite films with different TiO_2_ concentrations.

For manufacturing PP microporous membranes, the stretching strategy also greatly affects the porous structure and thus the final performance of the membranes [25]. Therefore, it is important to investigate the deformation behavior of the composite films to attain a deep insight into the pore formation during stretching. Figure 4 compares the stress–strain curves of the annealed PP and PP/TiO_2_ composite films with different TiO_2_ concentrations. Similar to that of the virgin PP film, the stress–strain curves of all PP/TiO_2_ composite films show two distinct yields at strains of about 10%–30% and around 80%–100%, respectively. Moreover, it is observed that no apparent necking phenomenon happens during stretching for all films. Such behavior is also referred to as hard elastic behavior, which is closely connected to the presence of a row-nucleated lamellar structure in the crystalline morphology of the as-prepared films [36]. It is claimed that the first and second yields correspond to the void creation in the amorphous region and the occurrence of the lamellar fragmentation, respectively, and the deformation between the two yields is related to the lamellar separation. The result is therefore indicative of that lamellar separation occurs when the PP/TiO_2_ composite films are subjected to uniaxial tensile stress. On the other hand, it can be observed that the yield stresses of the PP/TiO_2_ composite films show a declining tendency as the TiO_2_ content increases. Similar phenomena have been reported by other researchers for particulate-filled polymer composites, and it is mainly attributed to the occurrence of debonding of the filler from the polymer matrix [37,38]. In this work, no surface modification was carried out on the submicron-sized TiO_2_ particles, thus leading to a weak interfacial interaction in favor of debonding. Consequently, it suggests that both the lamellar separation and the debonding of the TiO_2_ particles from the PP matrix take place when uniaxially stretching the annealed PP/TiO_2_ composite films, which is verified by the SEM observations that follow.

### 3.3. Microstructure and Properties of the PP/TiO_2_ Composite Membranes

**Figure 5:**
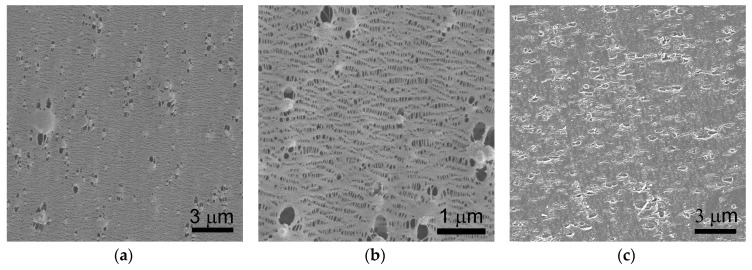
(**a**,**b**) Surface and (**c**) cross-section morphologies of PP/TiO_2_ composite membranes with 40 wt % TiO_2_.

Given the similar deformation behaviors among the PP and PP/TiO_2_ composite films, PP/TiO_2_ microporous membranes were prepared following an optimized stretching strategy of 35% cold extension and 60% hot extension used for the virgin PP film as we previously reported [24]. Figure 5a,b show the surface morphologies of the PP/TiO_2_ composite membranes loaded with 40 wt % TiO_2_. Similar to the commercial PP microporous membrane, the PP/TiO_2_ composite membrane possesses a large quantity of slit-like shaped micropores with a typical dimension of about 100 nm in length and 30–40 nm in width (analyzed by the image analysis software). This type of pore might be well connected with the plentiful oriented stacked lamellae in the composite film, which can be separated when the film is subjected to external stress, thus leading to the voids. In addition, another type of micropore with a larger size ranging from 200 to 500 nm can be found homogeneously distributed in the PP/TiO_2_ composite membranes. Specifically, these larger micropores generally appear nearby TiO_2_ particles, which can be reasonably attributed to the interfacial debonding between the TiO_2_ particles and PP matrix when the film is subject to external stress [39]. The presence of a large amount of micropores induced by the interfacial debonding can also be observed from the cross-section morphology as shown in Figure 5c.

**Figure 6:**
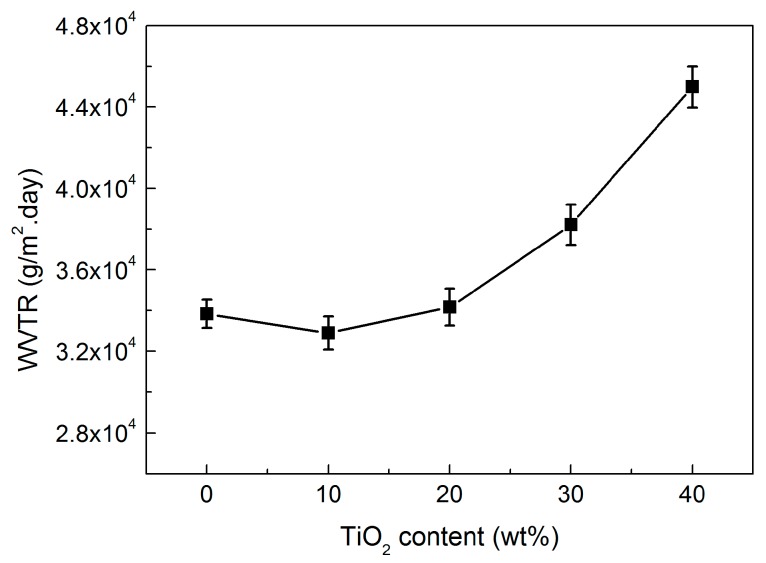
Water vapor transmission rate (WVTR) of the PP/TiO_2_ composite membranes as a function of TiO_2_ content.

Figure 6 shows the water vapor permeability of the microporous PP/TiO_2_ membranes as a function of TiO_2_ content. Excitingly, except for an initially slight decline at 10 wt % compared to the virgin PP membrane, continuous improvements in the permeability can be found for the PP/TiO_2_ composite membranes when the TiO_2_ loading ranges from 10 to 40 wt %. The results could be understandable by taking consideration of the dual roles of TiO_2_ particles. On the one hand, incorporation of the TiO_2_ particles causes a slight decrease in the PP crystalline orientation (as shown in Figure 3), thus reducing the proportion of the micropores coming from the lamellar separation and its contribution to permeability. On the other hand, increasing the TiO_2_ content increases the amount of larger micropores originating from the interfacial debonding between the TiO_2_ particles and PP matrix, and this helps to improve permeability. For the PP/TiO_2_ composite membrane at high TiO_2_ loadings, the latter would play a dominant role and thus lead to the increase in permeability on the whole. In this work, the permeability of the PP/TiO_2_ composite membrane reaches about 4.5 × 10^5^ g/m^2^/day when the TiO_2_ loading is 40 wt %, which is enhanced by nearly 33% compared to that of the virgin PP membrane, with a value of 3.38 × 10^5^ g/m^2^/day.

**Figure 7:**
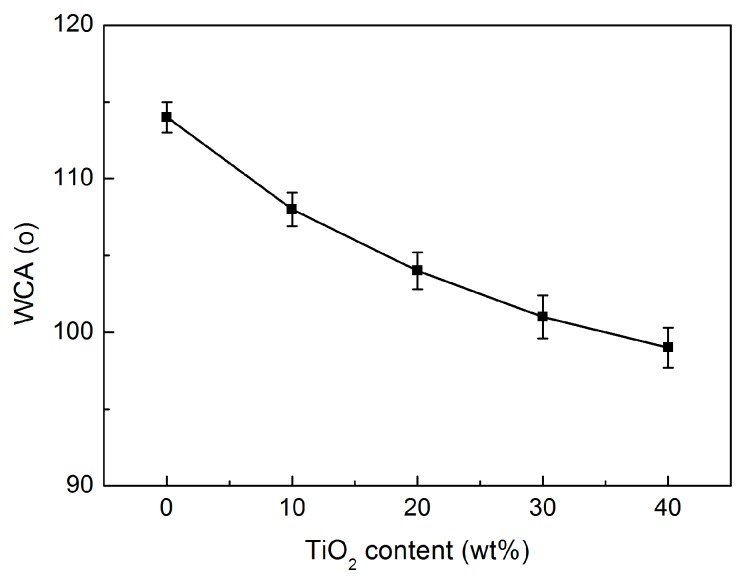
Water contact angle (WCA) of the PP/TiO_2_ composite membranes as function of TiO_2_ content.

Static water contact angle (WCA) can be used as an indicator to reflect the polarity of the membranes and thus the wettability with the liquid electrolytes. Figure 7 shows the water contact angle of PP/TiO_2_ composite membranes with different TiO_2_ concentrations. The virgin PP microporous membrane has an initial water contact angle as high as 114°, due to the hydrophobic nature as well as the surface roughness. The incorporation of TiO_2_ particles obviously decreases the WCA of the PP/TiO_2_ composite membranes, and the WCA decreases to 99° when the TiO_2_ loading is 40 wt %. The decrease of the WCA can be explained by the presence of many Ti–OH groups on the TiO_2_ particles at the surface, which could interact well with the water through van der Waals forces and hydrogen bonding [40].

**Figure 8:**
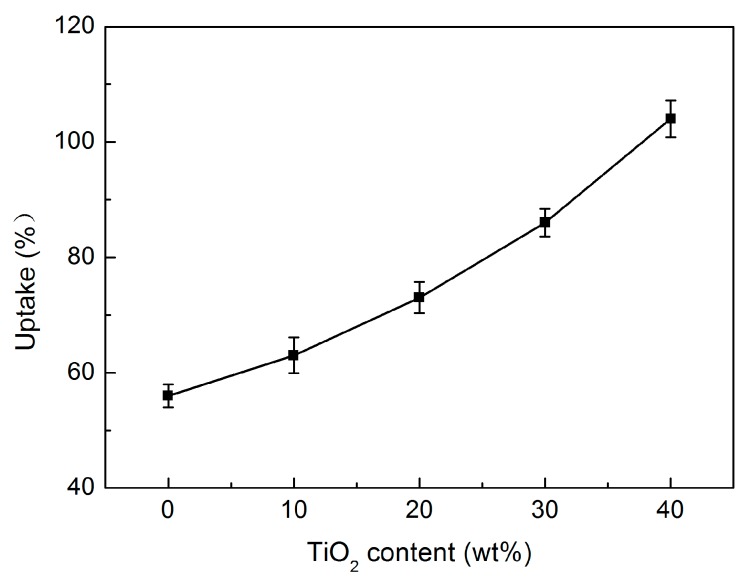
Electrolyte uptake of the PP/TiO_2_ composite membranes as a function of TiO_2_ content.

The electrolyte uptake of separators is considered an important parameter in battery performance. Figure 8 presents the electrolyte uptake values of PP/TiO_2_ composite membranes with different TiO_2_ concentrations. As expected, adding TiO_2_ particles cause obvious improvements in the electrolyte uptake for the PP-based membranes. It can be observed that the electrolyte uptake of the PP/TiO_2_ composite membranes increases from 56% to 104%, when the TiO_2_ loading ranges from 0 to 40 wt %. This means the electrolyte uptake capacity of the PP membrane can be virtually doubled by adding the TiO_2_ particles up to 40 wt %. It is believed that the presence of plentiful hydrophilic characteristics of the TiO_2_ particles, as well as some larger micropores from the interfacial debonding, both contribute to the improvement in the electrolyte uptake for the PP/TiO_2_ composite membranes compared to the virgin PP membrane.

The mechanical properties of the composite membranes with different TiO_2_ concentrations were measured as shown in Table 2. The virgin PP membrane has a tensile strength of about 118.4 MPa, which is similar to that of the commercial PP separators. It is found that incorporation of TiO_2_ particles slightly decreases the tensile strength from 118.4 to 104.6 MPa, reduced by about 12%, when the TiO_2_ content increases from 0 to 40 wt %. The result is different from previous work by Lei et al., which demonstrated that the mechanical strength of PP microporous membranes can be improved by the addition of nano-sized (20–60 nm) silica particles up to 10 wt % [28]. The difference may be attributed to the influence of the particle size. According to Fu et al., the composite strength is usually reduced with increasing particle loading when the particle size is larger than 80 nm [41]. Considering that the TiO_2_ particles have an average size of 0.5 μm and no surface treatment in this work, interfacial debonding between the filler and the PP matrix easily occurs, consequently leading to more voids in the stretched composite membranes and thus a reduction in the tensile strength compared to the virgin PP membrane. Besides, the elongation at break decreases slightly from 92.7% for the virgin PP membrane to 84.5% for composite membranes when the TiO_2_ content is up to 40 wt %. However, it should be mentioned here that all the composite membranes still possess excellent mechanical properties, which conform well to the battery separators [2].

## 4. Conclusions

In this work, the hydrophilic TiO_2_ particles of submicron size were used and compounded with the host PP pellets to improve the polarity and thus electrolyte uptake capability. The PP/TiO_2_ composites at different ratios were fabricated into microporous membranes following the melt extruding/annealing/stretching procedure, the same as that for the virgin PP. The results of the combination of DSC, XRD and FTIR suggest that the addition of TiO_2_ particles has little influence on the oriented lamellar structure of the PP-based composite films. Both the lamellar separation and interfacial debonding occur when the PP/TiO_2_ composite films are subjected to uniaxial tensile stress, consequently leading to two forms of micropores in the stretched membranes. Compared to the virgin PP membrane, the PP/TiO_2_ composite membranes at high TiO_2_ loadings show significant improvements in terms of the water vapor permeability, polarity, and electrolyte uptake capability.

## Figures and Tables

**Table 1:** Melting temperature (*T*_m_) and crystallinity (*X*_c_) for the polypropylene (PP) and PP/TiO_2_ composite films.

TiO_2_ content (wt %)	*T*_m_ (°C)	*X*_c_ %
0	164.4	41.8
10	164.1	41.5
20	164.8	42.0
30	163.9	42.1
40	164.2	41.6

**Table 2:** Mechanical properties of PP/TiO_2_ composite membranes with different TiO_2_ concentrations.

TiO_2_ content (wt %)	Tensile strength (MPa)	Elongation at break (%)
0	118.4	92.7
10	115.5	88.3
20	112.8	85.6
30	108.3	86.4
40	104.6	84.5
